# Lactate as a predictor of deterioration in emergency department patients with and without infection

**DOI:** 10.1186/cc13361

**Published:** 2014-03-17

**Authors:** A Oedorf, E Day, R Lior, AT Novack, E Shapiro, S Henning

**Affiliations:** 1 Beth Israel Deaconess Medical Center, Boston, MA, USA; 2 Soroka University Medical Center, Beersheba, Israel

## Introduction

The use of serum lactate level to risk-stratify emergency department (ED) patients with sepsis is widely used. Studies in nonsepsis populations also suggest its utility in predicting adverse outcomes. Whether lactate prognosticates equally across disease states is not clearly defined. This study compares the ability of lactate to identify patients at risk for deterioration (intubation, acute renal dysfunction, vasopressor use, or death) and mortality during hospitalization in infected and non-infected populations.

## Methods

A prospective, observational cohort study of ED adult patients presenting from 11 November 2012 to 1 February 2013 who had lactate measured and abnormal vital signs (hearth rate ≥130, respiratory rate ≥24, shock index ≥1, systolic blood pressure <90 mmHg). Patients with isolated atrial tachycardia, seizure, intoxication, or psychiatric agitation were excluded. Patients were stratified into three groups by lactate <2.5 (low), 2.5 to 4 (intermediate), and >4 mmol/l (high). Chi-square test for trend was used to compare outcome rates between lactate levels for each diagnostic category.

## Results

Of 1,152 patients identified, 366 were excluded and 298 did not have lactate measurements, leaving 488 for the analysis: 289 sepsis patients and 202 nonsepsis patients. Of these, 168 (34.4%) met the deterioration outcome, and there were 61 (12.5%) deaths. For infected patients, 46/342 (13.5%; 95% CI 10.2 to 17.5) low, 34/100 (34.0%; 95% CI 25.4 to 43.7) intermediate, and 20/46 (43.5%; 95% CI 30.2 to 57.8) high lactate patients suffered deterioration (*P *< 0.01). Likewise, 6/342 (1.6%; 95% CI 0.7 to 3.9) low, 19/100 (19.0%; 95% CI 12.4 to 27.9) intermediate, and 11/46 (23.9%; 95% CI 13.8 to 38.0) high lactate patients died during hospitalization (*P *< 0.01). For non-infected patients, 42/342 (12.3%; 95% CI 9.2 to 16.2) low, 13/100 (15.1%; 95% CI 7.6 to 21.1) intermediate, and 13/46 (28.2%; 95% CI 17.2 to 42.7) high lactate patients suffered deterioration (*P *= 0.01). In the regression models, lactate was strongly associated with deterioration for both infected (odds ratio (OR) = 1.94; 95% CI 1.4 to 2.3) and non-infected (OR = 1.48; 95% CI 1.14 to 1.91) groups. In regression models for mortality, lactate had better discrimination ability in infected (OR = 1.8; 95% Ci 1.4 to 2.3) patients than in the non-infected (OR = 1.11; 95% CI 0.85 to 1.47) patients.

## Conclusion

Lactate levels can be used to identify patients who are at increased risk of deterioration regardless of infection status. Lactate identifies high-risk patients for mortality in infected patients more strongly than in non-infected patients.

